# Quality Management Systems in the Ambulant Sector: An Analytical Comparison of Different Quality Management Systems

**DOI:** 10.3390/ijerph16030444

**Published:** 2019-02-02

**Authors:** Marcus Gumpert, Jens-Peter Reese

**Affiliations:** 1 Medical Faculty, Philipps-University of Marburg, Karl-von-Frisch-Strasse 4, 35043 Marburg, Germany; 2 Department of Quality Assurance/Quality Management, Association of Statutory Health Insurance Physicians in Saxony, Schützenhöhe 12, 01099 Dresden, Germany; 3 Coorsinating Center for Clinical Trials, Philipps-University of Marburg, Karl-von-Frisch-Strasse 4, 35043 Marburg, Germany; jenspeter.reese@kks.uni-marburg.de

**Keywords:** quality management systems, quality categories, outpatient quality assurance, evaluation in quality management, management systems for contract physicians

## Abstract

Background: Since 1 January 2004, all physicians, psychotherapists, and medical care centers that are under contract to statutory healthcare in Germany are obliged, according to § 135a Section 2 of the Fifth Social Security Statute Book, to introduce an intra-institutional quality management system. Methods: A total of 24 medical practices were chosen through random sampling. In total, there were 12 family physicians and specialist practices each and eight practices each per quality management system. The analysis was carried out with the help of three specially developed questionnaires (physician, employee, and patient). A total of 26 quality categories with different questions were available in the three survey groups (physicians, employees, and patients). The Kruskal–Wallis test checked the extent to which the different scores between the quality management systems were significant and effective for specialists or family physicians. Results: ”Quality and Development in Practices (QEP)” had the highest average score. Due to a specific family practitioner specialism, “Quality management in Saxony medical practices (QisA)” followed with good average scores. The individual quality categories in the quality management systems, such as the “range of services” or “allocation of appointments”, received the highest average scores among the specialists. In contrast, categories such as “telephone enquiries” and “external cooperation and communication” received the highest average scores among the family physicians. Conclusion: Differences in the evaluation of quality management systems and medical groups (specialists/family physicians) were found in the study. The reasons for these differences could be found in the quality categories.

## 1. Introduction

### 1.1. Background to the Study

Since 1 January 2004, all physicians, psychotherapists, and medical care centers that are under contract to statutory healthcare in Germany are obliged, according to § 135a Section 2 of the Fifth Social Security Statute Book (SGB V), to introduce and develop an intra-institutional quality management system [1,2,3,4].

Quality management systems differ significantly in terms of their structure and design, therefore, a direct comparison has not been possible in the past [5]. This analysis includes the most common systems in Saxony: Quality and Development in Practices (QEP), Quality management in Saxony medical practices (QisA), and DIN EN ISO 9001 [6,7,8,9,10].

QEP is a proprietary development of the National Association of Statutory Health Insurance Physicians (KBV) for practicing physicians and psychotherapists. It has been specially designed to meet the needs of the various specialist areas (e.g., radiology) as well as special features of outpatient care [9,10]. On the other hand, QisA is a proprietary development of the Association of Statutory Health Insurance Physicians in Saxony (KVS). While the KVS took up the idea of the QEPs in principle, it somewhat deviated from a theoretical and analytical adjustment in favor of a practical conversion [7]. DIN EN ISO 9001 defines the minimum requirements and standards for quality management systems according to the German Institute for Standardization and the International Organization for Standardization. However, the application of the system is difficult because the requirements have to be transferred to individual medical practices. Individual organizations also offer commercial support for DIN EN ISO 9001 [8].

Other quality management systems in Germany include the Cooperation for Transparency and Quality in Healthcare (KTQ) and other regional models such as the Association of Statutory Health Insurance Physicians Westphalia-Lippe Practice-Quality-Management (KPQM). KTQ is not suitable for this study because it is primarily aimed at hospitals [11], while KPQM is a regional quality management system analogous to QisA, which is used only marginally in Saxony [12].

Other quality management systems in Europe are the European Practice Assessment (EPA) or the European Foundation for Quality Management (EFQM). The EPA is aimed at general practitioners and does not take into account all quality categories given in the quality management guideline. The program is not suitable for specialist medical facilities; therefore, it is only conditionally suitable for consideration [13]. EFQM takes a holistic approach, similar to DIN EN ISO 9001. Due to the modular structure of the system, however, not all quality categories of the quality management guideline are available [14].

To date, no studies have dealt with the question of which quality management system has been evaluated to be the best. Likewise, it has not been analyzed to date whether individual quality categories are more suitable for specialists or family physicians. Individual publications have analyzed quality categories and attempted to standardize the content requirements and filter out collection categories. Furthermore, individual quality management systems have been examined in terms of content in studies and their influence on everyday practice. Moreover, the physician group has been analyzed [15,16,17].

Individual studies have dealt with the output of quality management systems after their introduction to individual institutions. Authors such as Kiess have analyzed quality management systems in special healthcare areas such as pediatrics [15], while other authors such as Kühlein et al. have examined how quality management systems have slowly gained acceptance in daily practice and have had a lasting influence on output [16].

Studies by Chiarini et al. analyze total quality management and the use of lean strategies to examine how these have improved patient satisfaction. However, this study does not specify whether there are demonstrable differences between the quality management systems used. Consequently, it is not possible to analyze whether different systems influence patient satisfaction. However, the present study is intended to address this issue [18].

The authors Manzanera et al. have also analyzed whether the results on quality assurance and safety culture in a facility are interrelated and whether the measures influence each other. Again, the quality management systems used were not compared [19].

The study by Tzelepis et al. examines the improvement of patient-oriented care for cancer patients. The practical structure is used to determine which aspects of cancer treatment were performed well and which areas needed improvement [20].

Unfortunately, all the studies primarily focus on quality management systems designed to achieve objectives for measuring patient satisfaction, the quality of the results in medical or rehabilitative measures, and general benefit (catalyst effect) through the use of management systems. A concrete system comparison, however, is missing completely so far [21,22,23].

### 1.2. The Aims of the Study

(1) Representation of the individual quality categories: The objective of this research is to filter out individual quality categories that permit a standardized comparison between the quality management systems, by using the list of objectives for the quality management guideline [1,2,24,25,26].

(2) Evaluation of the individual quality management systems: The objective of the study is to support the physicians in choosing their quality management system. The region of the Association of Statutory Health Insurance Physicians in Saxony in Germany has been chosen for this purpose. In the present paper, a cross-sectional study comparing three quality management systems was carried out.

(3) Evaluation of the individual quality categories according to specialists and family physicians: The study analyzes significant differences in the average scores between the individual quality management systems for specialists or family physicians.

(4) Supporting the physicians in the selection of a suitable quality management system: By providing information on how quality management systems are evaluated and how quality categories are developed, the study can help doctors and psychotherapists in choosing a system.

At the current state of research, there is no systematic decision tool available to doctors and psychotherapists.

To date, no analyses have been carried out for central quality management systems such as QEP. The physicians do not know which central quality categories comprise the quality management systems. Furthermore, there are no findings for specialists or family physicians.

The objective of this research is to filter out individual quality categories per quality management system that would permit a standardized comparison between the quality management systems, by using the list of objectives for the quality management guideline.

The study analyzes significant differences in the average scores (expected values) between the individual quality management systems for specialists or family physicians.

Doctors and psychotherapists, therefore, have the possibility for the first time to select from the multitude of systems the best-suited quality management system for their own practice. The medical practice can also align its interests and strengths with the specialist or family physician quality categories for a good overall assessment.

## 2. Methods

### 2.1. Design

The study analyzes significant differences in the average scores between the individual quality management systems for all physicians, and for specialists or family physicians.

Quality management systems [6,7,8,9,10]:

- DIN EN ISO 9001 (specialists/family physicians)
- Quality and Development in Practices (QEP) (specialists/family physicians)
- Quality management in Saxony medical practices (QisA) (specialists/family physicians)

### 2.2. Study Groups

A family physician is a resident or employed physician who is usually the first point of contact for the patient with medical problems. He has to be within the framework of the family physician model. He coordinates the subsequent further treatment with individual specialists.

A specialist is a physician with a recognized further training in a medical field.

Questionnaire ”physicians” includes the two groups of physicians (specialists and family physicians).

Questionnaire ”employee” includes all non-medical employees working in a medical practice.

Questionnaire ”patient” includes all people who are regularly being treated in the medical practice.

### 2.3. Setting

The study has been carried out in the area of the Association of Statutory Health Insurance Physicians in Saxony, because the outpatient-contracted medical sector guarantees a high qualitative and quantitative use of quality management systems in the practices. The population includes all physicians who are established in a practice. A total of 24 medical practices (random sample) were available. First of all, two primary units (one specialist group each for specialists and general practitioners) were determined from the basic population of all active physicians. In the next step, a quota sample was drawn for each primary unit (specialist and general practitioner). The three most common quality management systems (QEP, QisA and DIN EN ISO 9001) were selected. A total of 12 specialist and family doctor practices and eight practices per quality management system were available. By using the Kruskal–Wallis test, the quality management systems were analyzed in an open-ended manner with the aim to check the extent of differences in the evaluation of the quality management systems and whether individual quality categories are more likely to be characterized by specialists or by family physicians.

### 2.4. Data Collection

The analysis was carried out with the help of three self-developed questionnaires (physician, employee, and patient). The individual questions were developed independent of a quality management system. The basis was formed by the specifications of the quality management guideline given by the Joint Federal Committee, which describes the concrete content of each quality category. The contents of the quality management guideline were used to present the individual quality categories and to create questions for the questionnaires. A total of 26 quality categories with 40 different questions were available. The focal points of the survey were identical in terms of content; however, they were adapted to the respective survey group (physicians, employees, and patients). For example, in the quality category “personnel planning and employee discussions”, the employees were directly asked about the extent to which delegation and feedback discussions take place. The patient was asked indirectly whether a distribution of tasks and delegation is apparent. This ensured that the questionnaires were structurally identical in their response behavior. We deliberately included three survey groups (physicians, employees, and patients) to obtain robust results and to ensure that the results were not influenced by any one group. Additional contents of the quality management systems were deliberately not considered, because they did not result from the legal norms. This ensured that all quality categories were present in all quality management systems.

Through independent questionnaires, the connection to a quality management system could be excluded. The questionnaires feature average scores from one to four, with four (very good) being the highest and one (very poor) the lowest score. Each practice received 75 patient questionnaires for all doctors/employees. Authors such as MacCallum et al. recommend a sample size (number of patient questionnaires) of at least 100 from statistical studies. Taking into account a possible low response rate of 10-15%, a conscious decision was taken to issue a total of 1901 questionnaires. The questionnaires were sent to the institutions along with explanations about the study. The questionnaires were handed out by the physician or practice staff at the time of registration. The individual quality categories are shown in Table 1. A pre-test was carried out with three institutions to check the comprehensibility of the questionnaires [27,28,29].

### 2.5. Data Analysis

A full survey was not suitable since there are approximately 8250 doctors and psychotherapists in Saxony and since quality management systems are yet to be introduced. The results are representative of the facilities since various types of practice were taken into account, including individual and joint practices as well as urban and rural regions.

Analogous studies, such as those conducted by Health Foundation and TÜV SÜD (technical-testing organization), examined 191 questionnaires from institutions. In this study, a total of 1646 questionnaires with a total of 26 question complexes were available for evaluation from more than 24 institutions. Due to this scope, the prerequisites for the application of the individual test procedures could also be ensured [36,37].

For the analysis of the data, the arithmetic mean was calculated in the individual question complexes. Subsequently, a Kruskal–Wallis test was used to determine whether the quality management systems for physicians and for specialist physicians/family doctors differed with statistical significance on average. Taking into account the costs of the individual quality management systems and their introduction, the effect strength was finally analyzed according to η^2^ and Cohen’ s d. Using these findings, the institutions could decide which system should be used [36,37,38,39,40,41].

## 3. Results

In all, 27 physicians, 74 employees, and 1,800 patient questionnaires were issued. The response rate was over 85%. Finally, 27 physicians, 74 employees, and 1545 patient questionnaires were returned.

### 3.1. Differences in the Average Scores of the Quality Management Systems

**Figure 1:**
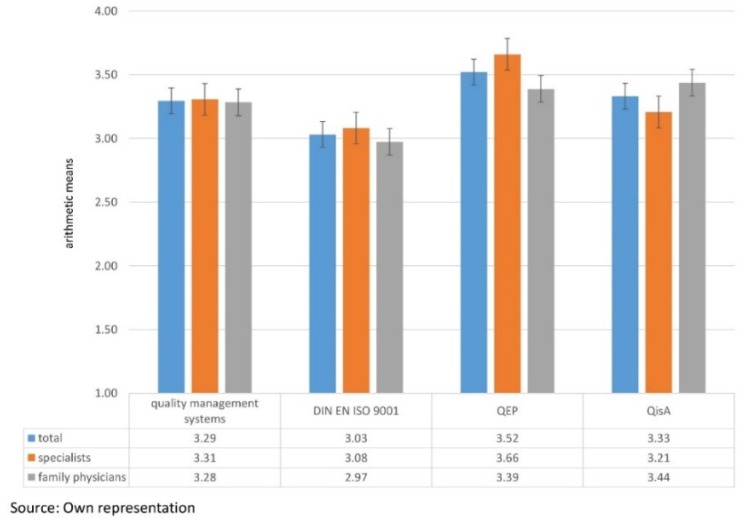
Average scores of quality management systems. Figure 1 shows the average scores of quality management systems. The questionnaires featured scores from one to four, with four (very good) being the highest and one the lowest (very poor) score.

Figure 1 shows the individual mean average scores for the quality management systems. The questionnaires featured average scores from one to four, with four (very good) being the highest and one (very poor) the lowest score. This revealed that the overall grading of quality management systems was good with an average score of 3.29. In the analysis, QEP had the highest average score with 3.52. This was followed by QisA with 3.33 and DIN EN ISO 9001 with 3.03. Apart from the overall results, Figure 1 also shows the scores for specialists and family physicians. For DIN EN ISO 9001 (specialists: 3.08 and family physicians: 2.97) and QEP (specialists: 3.66 and family physicians: 3.39), the average scores were higher for the specialists than for the family physicians. QisA revealed the opposite result. For QisA, the family physicians obtained a higher average score with 3.44 than the specialists with 3.21. From the results of this study, QEP could be recommended for specialists, while QisA, with the average score of 3.44, could be considered as an alternative for family physicians [6,7,8,9,10].

### 3.2. Differences in the Average Scores of Quality Management Systems Among Specialists and Family Physicians

Table 2 shows the highest average scores for QEP (specialists) (dark grey), QisA (family physicians) (grey), and DIN EN ISO 9001 (specialists) (light grey).

The highest average scores were recorded in QEP (specialists) (red line) in the categories ”range of services (Ø = 3.79)”, ”allocation of appointments (Ø = 3.69)”, “treatment pathways and guidelines (Ø = 3.93)”, “disease-specific measures (Ø = 3.88)”, “patient education (Ø = 3.68)”, “patient safety, risk- and error-management (Ø = 3.65)”, “personnel planning and employee discussions (Ø = 3.56)”, “maintenance and service (Ø = 3.83)”, “occupational safety (Ø = 3.78)”, “hygiene and cleaning (Ø = 3.78)”, “services and interventions (Ø = 3.88)”, and “health promotion and prevention (Ø = 3.88)”. Moreover, these quality categories were dominated by specialists, because all scores—regardless of the quality management system—were better for the specialists than for the family physicians. This also applied to the quality categories “continuing professional development and qualification (Ø = 3.81)”, “infrastructure (Ø = 3.82)”, and “procurement and storage (Ø = 3.81)”, even though the highest scores were with DIN EN ISO 9001 (specialists) (blue line). The quality categories “treatment pathways and guidelines (Ø = 3.93)”, “disease-specific measures (Ø = 3.88)”, “maintenance and service (Ø = 3.83)”, “services and interventions (Ø = 3.88)”, and “health promotion and prevention (Ø = 3.88)” were dominated by QEP (specialists), reaching almost the best rating of 4.

By contrast, the quality categories “telephone enquiries (Ø = 3.80)”, “external cooperation and communication (Ø = 3.80)”, “emergency management (Ø = 3.80)”, “maintaining patient records (Ø = 3.90)”, “initial patient information (Ø = 3.77)”, “confidentiality and professional secrecy (Ø = 3.86)”, “organizational structure (Ø = 3.80)”, “quality objectives (Ø = 3.80)”, “quality management-practice handbook (Ø = 3.75)”, and “prescriptions (Ø = 3.71)” received the highest average scores with QisA (family physicians) (green line). These quality categories were dominated by family physicians, because all scores—regardless of the quality management system—were better for family physicians than for specialists.

### 3.3. Evaluation of the Questionnaire Groups

**Figure 2:**
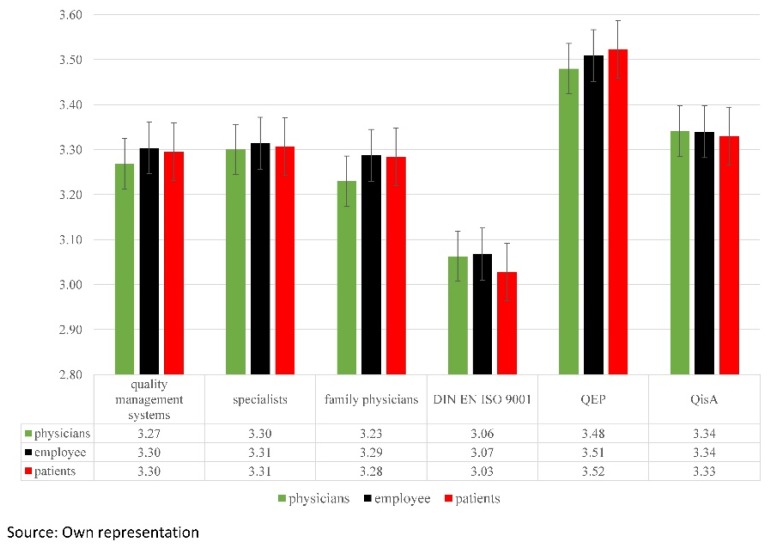
Average scores of questionnaire groups. Figure 2 shows the average scores of quality management systems for the individual survey groups. The questionnaires feature scores from one to four, with four (very good) being the highest and one the lowest (very poor) score. No significant deviations could be detected.

Figure 2 shows the individual arithmetic averages shown in Figure 1 and Figure 2 for all quality categories. In addition, the individual arithmetic averages for physicians, employees, and patients are given for each quality management system.

There were no significant differences between the three survey groups (physicians, employees, and patients) in the individual quality management systems. The survey of three groups had the primary goal of obtaining a general and representative result of the practice. The intention was, in comparison to other survey studies, to avoid an excessive weighting of the physician or patient results.

### 3.4. Kruskal–Wallis Test between the Individual Quality Management Systems and Doctor Groups

In Table 3, the Kruskal–Wallis test was used to determine the extent to which the different average scores between the quality management systems and quality categories were significant [29,35,36,37,38,39,40,41].

The dark grey fields in Table 3 depict the highest mean rank sums. This confirms the individual average scores in Figure 1 and Table 2. In principle, the highest mean rank sums in the individual quality categories were always with specialists or family physicians—regardless of the quality management system. The middle-grey and light-grey areas defined the second and third best average scores.

The highest mean rank sum in the quality category “range of services” was found for QEP (specialists), followed by QisA (specialists) and DIN EN ISO 9001 (specialists). This also applied to the quality categories “allocation of appointments”, “treatment pathways and guidelines”, “disease-specific measures”, “patient education”, “patient safety, risk- and error-management”, “personnel planning and employee discussions”, “maintenance and service”, “occupational safety”, “hygiene and cleaning”, “services and interventions”, and “health promotion and prevention”. The quality categories “continuing professional development and qualification”, “infrastructure”, and “procurement and storage” had the highest average scores among the specialists (but by DIN EN ISO 9001). All results were highly significant with *p* < 0.000001.

Table 3 illustrates that for the quality categories “telephone enquiries”, “external cooperation and communication”, “emergency management”, “maintaining patient records”, “initial patient information”, “confidentiality and professional secrecy”, “organizational structure”, “quality objectives”, “quality management-practice handbook”, and “prescriptions”, generally the highest mean rank sum was with QisA (family physicians). The second and third best scores were observed for QEP (family physicians) and DIN EN ISO 9001 (family physicians).

Based on the analysis using the Kruskal–Wallis test, the individual quality categories were subject to specialist or family physicians’ criteria, which provides the offices with a choice for the “right” quality management system or allows an individual system analysis to be allocated to each medical practice by means of the quality categories.

Finally, the results in Table 3, which lists the three quality management systems, medical groups, and quality categories, are to be analyzed based on the effect size by η^2^ and their transformation to the effect strength according to Cohen’ s d. Using Cohen’ s d effect size, the significant mean differences between different quality management systems and specialists/family physicians could be evaluated in terms of their practical relevance [42,43,44]. An ETA-squared value between 0.060 and 0.110 means a medium effect size and greater than 0.140 means a great effect size. A Cohen’s d value between 0.5 and 0.7 means a medium effect size and greater than 0.8 means a great effect size [42]. It was possible to demonstrate a large effect size in addition to the highly significant results for all test methods.

In addition, the Bonferroni method was used to neutralize the alpha error cumulation in multiple comparisons. For n independent hypotheses of a dataset, the statistical significance was indicated separately, which is comparable to a test with 1/*n*^th^ of the significance level [29].

## 4. Discussion

The response rate was 85%. The reasons for this high level are the extensive information material and the breakdown of the questionnaires into physician, employee, and patient types. The highest level of acceptance was achieved when the questionnaires were personally handed out by the doctor or the practice staff to the patients when they registered.

### 4.1. Representation of the Individual Quality Categories

A total of 26 quality categories were analyzed, which had a lasting influence on the evaluation of the quality management systems. Furthermore, individual quality categories showed better results with both specialists and family physicians, irrespective of which quality management system is considered. In the following section, we present the quality categories with a very high or very low average score and establish a ranking (see Table 2).

The QEP has the following quality categories:

- treatment pathways and guidelines (Ø = 3.93)
- disease-specific measures (Ø = 3.88)
- maintenance and service (Ø = 3.83)
- services and interventions (Ø = 3.88)
- health promotion and prevention (Ø = 3.88)

QisA has the following quality categories:

- maintaining patient records (Ø = 3.90)
- confidentiality and professional secrecy (Ø = 3.86)

DIN EN ISO 9001 has the following quality categories:

- continuing professional development and qualification (Ø = 3.81)
- infrastructure (Ø = 3.82)
- procurement and storage (Ø = 3.81)

### 4.2. Evaluation of the Individual Quality Management Systems

When analyzing the individual results, it becomes obvious that the quality management system QEP hasd the best average scores at 3.52, followed by QisA at 3.33 and DIN EN ISO 9001 at 3.03. When classifying the quality management systems in terms of specialists and family physicians, the same pattern was revealed for specialists. With an average score of 3.66, QEP had the best result. This is followed by the QisA and DIN EN ISO 9001 systems. The family physicians group reflected a different pattern. Here, QisA received the highest average score. The reasons for the sequence and evaluation of the individual quality management systems were based on the individual quality categories. The overall result was based on quality categories of varying strength and weakness.

### 4.3. Evaluation of the Individual Quality Categories

The following indicators lead to the best average scores for QEP specialists (Table 2) [6,7,8,9,10]:

- range of services
- allocation of appointments
- treatment pathways and guidelines
- disease-specific measures
- patient education
- patient safety, risk- and error-management
- personnel planning and employee discussions
- service and maintenance
- occupational safety
- hygiene and cleaning
- services and interventions
- health promotion and prevention

In addition to their high significance, the quality categories mentioned had a high effect strength [36,37,38].

The QEP system features many pattern documents and process flow diagrams. These pattern documents and process flow diagrams offer more detailed, additional explanations and more pronounced in the quality categories “range of services”, “allocation of appointments”, “treatment pathways and guidelines”, “disease-specific measures”, “patient education”, and “patient safety, risk-, and error-management” than in QisA or DIN EN ISO 9001. QEP provides supporting documentation, for example an “allocation of appointment”. In this, the individual appointments are consciously classified as consultations for prevention or check-ups. With QisA, the “allocation of appointments” during initial patient information has a negative effect. The lack of pattern documents is a disadvantage in DIN EN ISO 9001. QisA lacks specialist depth, particularly in the categories of “personal planning and employee discussions”, “service and maintenance”, and “occupational safety”.

QEP features many samples, such as discussion guidelines or hygiene plans under “hygiene and cleaning”, “services and interventions”, and “health promotion and prevention”. The other systems do not support the practices with templates, but only explain their content. QEP and QisA feature stock protocols for “hygiene and cleaning”. These documents are more comprehensive in comparison to DIN EN ISO 9001. The “services and interventions” is not supported at all in QisA and DIN EN ISO 9001.

The best average score for the family physicians is found with QisA (Table 2) [6,7,8,9,10]:

- telephone enquiries
- external cooperation and communication
- emergency management
- maintaining patient records
- initial patient information
- confidentiality and professional secrecy
- organizational structure
- quality objectives
- quality management-practice handbook
- prescriptions

In addition to their high significance, the mentioned quality categories have a high effect strength [36,37,38].

A key task of the family physicians is to communicate with specialists to coordinate diagnostics and therapy. The “telephone enquiries” and “external cooperation and communication” ratings, therefore, are highly significant and effective. The system lists the requirements in more detail and with a better structure than the other systems.

QisA features a process plan for emergency treatments to support the medical practice (“emergency management”). For QEP, emergencies are explained in general terms only. Both systems feature a checklist for the emergency cases, which is missing with DIN EN ISO 9001. The tools in QisA resulted in a high score of 3.80.

The high score for QisA at “maintaining patient records” in QisA (3.90) was due to the comprehensive explanations on the creation and maintenance of the patient records.

In QisA, pattern examples and variants are explained for consultation and availability. The QEP and DIN EN ISO 9001 systems lack sample templates and supporting materials for “initial patient information” and “confidentiality and professional secrecy”.

In QisA, the “organizational structure” and the “quality management-practice handbook” are represented in a compressed and simple form. It was helpful that a sample organization chart, which includes processes and responsibilities, has already been compiled.

Despite the small size of the random sample and the initial analysis of the differences in quality management systems, the results were highly significant and highly effective.

### 4.4. Evaluations of the Questionnaire Groups

The analysis of the individual survey groups showed for all quality categories that there were no significant differences between the assessments of doctors, employees, and patients. This prevented any survey group from having a significant positive or negative influence on the overall result and ensured that there was no uniform evaluation picture.

### 4.5. Supporting the Physician in the Selection of a Suitable Quality Management System

These results illustrate that there were fundamental differences in the average score of the various quality management systems. This statement was important for providing a decision tool for individual medical practices to determine which quality management system was most suitable for specialists or family physicians.

Due to the highly significant result between the quality management systems and the extent of the effect size, the QEP system can be recommended for introduction in a specialist practice. The QEP has detailed documents in several quality categories including appointments, diagnostic, therapeutic services, and emergency, hygiene, or equipment management.

### 4.6. Limitations of the Research

The strength of the study can be seen in two aspects. On the one hand, 26 quality categories could be developed. From these categories, physicians and psychotherapists now have the opportunity to determine which of the 26 quality categories are most suitable for their institution.

On the other hand, the Kruskal–Wallis test provided new results on which quality management systems received the best average scores. The analysis was also extended to include specialists and family physicians.

With this knowledge, the physician and psychotherapy facilities can, for the first time, make a conscious choice regarding a suitable quality management system. The institution can select a suitable quality management system on the basis of its interests and strengths via the quality categories.

Furthermore, with 1646 questionnaires, a very robust survey was available, which was distributed among the 26 quality categories and ensured significant results. Due to the introduction of the quality management guideline, only institutions with quality management systems have been included in the survey.

The limited presentation of the three quality management systems can be seen as a weakness. The number of quality management systems and doctor groups should be increased in follow-up studies.

### 4.7. Practical Implications

The analysis showed that QEP is best suited for specialists, while QisA is more optimal for general practitioners.

It was also observed that individual quality categories are more specialist specific or family doctor specific. With this knowledge, the medical practices can check which quality categories are of interest for the establishment and select the quality management system purposefully. Moreover, state institutions or private companies can also use these findings to sustainably develop and improve their quality management system.

### 4.8. Further Research Questions

This study was the first to provide a presentation of uniform quality categories. Furthermore, significant and effective results could be identified in the evaluations of the quality management systems. Thus, for the first time, practices have a conscious decision-making aid. Furthermore, demonstrable differences between family physicians and specialists can be seen.

To date, individual studies have dealt with the standardization of quality management systems with regard to the quality categories in the quality management directive. These include studies by Sieger on the definition of a DIN EN set of rules or the KBV practice manual [9,14,15,16,17].

Other studies deal with the output of the quality management systems after their introduction to the individual institutions. Authors such as Kiess analyzed quality management systems in special care areas such as pediatrics [15], while other authors such as Kühlein et al. examined how quality management systems have slowly gained acceptance in everyday practice and influenced the output with lasting effects [16].

Unfortunately, studies primarily present quality management systems as a means of achieving objectives for measuring patient satisfaction, the quality of results in medical or rehabilitative measures, and general benefits (catalyst effect) from the use of management systems. However, a concrete system comparison is completely missing at present [21,22,23].

## 5. Conclusions

Finally, we would like to consider and summarize the individual research questions.

We were able to develop individual quality categories that allow a standardized comparison between the quality management systems. This enables comparisons to be made with other systems and doctor groups.

The study showed that there were differences in the evaluation of quality management systems. The QEP had the highest rating, followed by the QisA and DIN EN ISO 9001 systems.

In this context, a specialist quality category in each system received a higher average score with the specialists. Family physician quality categories in each system received a higher average score with the family physicians [9,10,45,46].

For further analyses, the individual quality management systems should be extended by including other Europe-wide systems. A breakdown by specific specialist physicians should be made for further analysis to see the extent to which these results are still valid.

## Figures and Tables

**Table 1:** Quality categories.

Quality Categories	Content of the Questionnaires
range of services	The practises detail their specific “range of services”. This is determined by their specialist status, their focus and special features of their practise [30].
allocation of appointments	According to § 17 Section 1 Federal Master Treaty, the physicians must offer sufficient working hours for appointments (at least 20 hours per week) [9].
telephone enquiries	The employees must know which patient enquiries may be answered by the physician or the personnel.
treatment pathways and guidelines	The “treatment pathways and guidelines” should be tools to support decisions that objectify and systematize the diagnostic and therapeutic questions [31].
disease-specific measures	In complex medical discussions, it is useful to support the patient with written documents. Information may be offered during the treatment or in the waiting room for this purpose [32,33].
external cooperation and communication	The family physician plays a coordinating role between the specialists in the treatment of the patient [9].
emergency management	Patients with health-related disorders must be identified and treated quickly [9].
maintaining patient records	Each patient record must have a clear structure. It must reveal the most important data about the patient [9].
patient education	This category investigates how the patient is educated about medical measures [9].
initial patient information	The patient record must not contain any discriminatory remarks about the “initial patient information” [34].
patient safety, risk- and error-management	Drugs are prescribed in medical practises, diagnostic investigations are performed and basic medical tasks are delegated [9,35].
confidentiality and professional secrecy	Due to the sensitive situation of the patient, their privacy must be maintained at all times.
personnel planning and employee discussions	Practice employees should be assigned clearly defined tasks.
continuous professional development and qualification	Targeted continuous professional development and training for the practise employees promotes improvement to procedures and processes.
organizational structure	The quality management guideline describes the “organizational structure” [9].
infrastructure	It is mandatory that medical practises are accessible to patients with walking aids or wheelchairs. On the other hand, access options for buggies are recommended for paediatric practises [9].
service and maintenance	It should be ensured that technical equipment is used by qualified personnel. Physicians and employees are supported in the handling of the equipment by operating instructions [9,26].
occupational safety	The practise management must create a safe working environment for patients and employees [9].
hygiene and cleaning	The guidelines must be used for the implementation of the hygiene specifications [9].
quality management system	A systematic system is important for the many different procedures and processes in a practise [9].
quality objectives	“Quality objectives” can be classified into primary, qualitative and quantitative objectives.
quality management-practise handbook	The practise handbook should define in writing the rules of the practise and quality-relevant aspects, such as treatment paths. The handbook should be published so that the patient can submit an assessment for the practise.
prescriptions	“Prescriptions” must always be filled correctly [31].
services and interventions	Certain services require that the physicians and employees fulfil certain quality requirements.
health promotion and prevention	Preventive measures promote the early detection and treatment of diseases.
procurement and storage	There are many suppliers where practises can purchase consumables and drugs. Selection criteria may be the price or the reliability [9].

Source: Own representation.

**Table 2:** Average scores of the quality management systems by quality categories and groups.

Fragenkomplex	DIN EN ISO 9001	QEP	QisA		DIN EN ISO 9001		QEP		QisA	
					Specialists	Family Physicians	Specialists	Family Physicians	Specialists	Family Physicians
range of services	2.90	3.43	3.07		3.08	2.69	3.79	3.08	3.09	3.06
allocation of appointments	2.73	3.39	3.03		2.80	2.65	3.69	3.11	3.07	3.00
telephone enquiries	2.64	3.54	3.40		2.59	2.70	3.52	3.56	2.94	3.80
treatment pathways and guidelines	3.07	3.61	3.20		3.09	3.04	3.93	3.30	3.31	3.10
disease-specific measures	2.87	3.49	3.13		3.01	2.70	3.88	3.11	3.28	3.00
external cooperation and communication	3.29	3.68	3.63		3.25	3.33	3.62	3.73	3.42	3.80
emergency management	3.15	3.54	3.50		3.05	3.26	3.52	3.57	3.14	3.80
maintaining patient records	3.26	3.76	3.68		3.22	3.32	3.72	3.79	3.42	3.90
patient education	2.86	3.37	3.07		2.97	2.73	3.68	3.08	3.13	3.02
initial patient information	2.97	3.56	3.45		2.94	3.00	3.51	3.61	3.06	3.77
patient safety, risk- and error-management	3.15	3.47	3.27		3.25	3.05	3.65	3.29	3.34	3.22
confidentialy and professional secrecy	3.30	3.74	3.72		3.29	3.30	3.71	3.77	3.55	3.86
personnel planning and employee discussions	2.81	3.32	3.12		2.89	2.60	3.56	3.06	3.23	3.00
continuous professional development and qualification	3.46	3.49	3.47		3.81	2.50	3.64	3.31	3.47	3.47
organizational structure	3.24	3.61	3.55		3.22	3.26	3.59	3.63	3.25	3.80
infrastructure	3.43	3.60	3.47		3.82	2.99	3.71	3.49	3.50	3.43
service and maintenance	3.04	3.43	3.26		3.09	2.99	3.83	3.06	3.27	3.25
occupational safety	2.89	3.48	3.21		2.94	2.83	3.78	3.20	3.22	3.20
hygiene and cleaning	3.18	3.56	3.48		3.24	3.00	3.78	3.31	3.50	3.47
quality management system	2.68	3.18	3.17		2.56	3.00	3.06	3.31	3.07	3.27
quality objectives	2.86	3.32	3.30		2.81	3.00	3.22	3.44	2.80	3.80
quality management-practise handbook	2.75	3.30	3.27		2.68	2.83	3.10	3.49	2.70	3.75
prescriptions	3.07	3.39	3.37		3.03	3.12	3.25	3.53	2.97	3.71
services and interventions	2.98	3.55	3.23		3.11	2.84	3.88	3.24	3.24	3.21
health promotion and prevention	3.01	3.52	3.14		3.10	2.90	3.88	3.17	3.19	3.10
procurement and storage	3.38	3.50	3.42		3.81	2.20	3.67	3.31	3.43	3.40

Source: Own representation.

**Table 3:** Kruskal–Wallis test.

Questions	Mean Rank Sum						Chi-Squared	[Df]	Asymptotic Significance	Effect Size According to H^2^	Effect Size According to Cohen’s d
	DIN Specialists	DIN Family Physicians	QEP Specialists	QEP Family Physicians	QisA Specialists	QisA Family Physicians					
range of service	783.99	444.61	1389.70	753.70	834.54	744.49	595.7080	5.00	<0.000001	0.390	1.492
allocation of appointments	654.13	589.99	1222.88	846.82	863.99	778.58	348.5896	5.00	<0.000001	0.208	1.025
telephone enquiries	461.32	525.03	1,011.20	1033.89	657.91	1191.81	665.7452	5.00	<0.000001	0.400	1.633
treatment pathways and guidelines	669.65	620.41	1347.59	839.18	844.61	661.58	458.6346	5.00	<0.000001	0.290	1.231
disease-specific measures	685.19	459.40	1424.28	786.37	932.90	682.40	653.1031	5.00	<0.000001	0.446	1.607
external cooperation and communication	535.66	595.27	907.76	1029.09	719.64	1105.46	379.3159	5.00	<0.000001	0.227	1.083
emergency management	510.12	692.06	929.11	974.22	607.90	1177.00	439.7436	5.00	<0.000001	0.263	1.195
maintaining patient records	557.46	666.21	912.89	994.31	722.56	1052.85	338.7056	5.00	<0.000001	0.202	1.006
patient education	682.71	598.29	1300.85	814.24	845.78	726.17	365.5875	5.00	<0.000001	0.218	1.057
initial patient information	487.72	511.60	996.02	1077.24	591.19	1211.16	690.1791	5.00	<0.000001	0.415	1.684
patient safety, risk- and error-management	775.39	646.65	1,130.37	792.82	847.23	761.64	163.2550	5.00	<0.000001	0.180	0.651
confidentialy and professional secrecy	634.01	645.21	894.53	937.65	790.22	1006.79	211.1905	5.00	<0.000001	0.140	0.755
personnel planning and employee discussions	44.15	36.20	68.67	49.94	56.07	48.07	12.9925	5.00	<0.000001	0.136	0.568
continuous professional development and qualification	64,07	20.35	57.72	44.22	49.83	48.23	23.5778	5.00	<0.000001	0.272	0.917
organizational structure	656.20	692.80	901.90	930.22	665.62	1056.30	226.2623	5.00	<0.000001	0.140	0.787
infrastructure	1028.68	551.02	954.15	827.57	829.84	740.13	214.7406	5.00	<0.000001	0.150	0.763
service and maintenance	712.82	650.59	1228.99	711.75	829.42	816.99	292.7535	5.00	<0.000001	0.180	0.919
occupational safety	663.90	634.26	1186.41	793.56	845.51	826.22	261.3655	5.00	<0.000001	0.155	0.857
hygiene and cleaning	42.67	38.90	67.67	47.19	54.70	54.43	12.9622	5.00	<0.000001	0.138	0.567
quality management system	34.13	51.20	53.17	64.75	53.70	61.27	17.5136	5.00	0.003622	0.158	0.788
quality objectives	38.94	46.00	50.33	63.03	39.57	75.43	23.9098	5.00	<0.000001	0.192	0.927
quality management-practice handbook	535.23	617.64	765.84	979.35	537.39	1,129.99	484.3620	5.00	<0.000001	0.309	1.338
prescriptions	623.86	690.57	773.08	996.01	648.26	1,150.81	312.9589	5.00	<0.000001	0.186	0.957
services and interventions	709.44	581.24	1276.66	824.84	795.30	769.74	349.3415	5.00	<0.000001	0.208	1.026
health promotion and prevention	721.03	625.53	1338.52	814.92	787.17	689.05	402.2985	5.00	<0.000001	0.240	1.125
procurement and storage	64.57	22.50	57.83	43.28	47.57	49.03	22.5309	5.00	<0.000001	0.336	0.885
total	13,441.82	12,450.07	22,192.06	18,011.62	15,382.59	18,563.25	4130.8390	5.00	<0.000001	0.125	0.750

Source: Own calculation and [38].

## Data Availability

The datasets used and/or analysed during the current study are available from the corresponding author on reasonable request. The participating institutions were known. The surveys were anonymous.
